# Prescription opioid misuse and its relation to injection drug use and hepatitis C virus infection: protocol for a systematic review and meta-analysis

**DOI:** 10.1186/2046-4053-3-95

**Published:** 2014-09-02

**Authors:** Ashly E Jordan, Don Des Jarlais, Holly Hagan

**Affiliations:** 1 New York University College of Nursing, 726 Broadway, New York NY 10003, USA; 2 Center for Drug Use and HIV Research, 726 Broadway, New York NY 10003, USA; 3 Chemical Dependency Institute, Mount Sinai Beth Israel, 160 Water Street, 24th floor, New York NY 10038, USA

**Keywords:** Prescription opioid misuse, Initiation of injection drug use, Epidemiology, Hepatitis C, Systematic review

## Abstract

**Background:**

The production, prescription, and consumption of opioid analgesics to treat non-cancer pain have increased dramatically in the USA in the past decade. As a result, misuse of these opioids has increased; overdose and transition to riskier forms of drug use have also emerged. Research points to a trend in transition to drug injection among those misusing prescription opioids, where clusters of acute hepatitis C virus (HCV) infection are now being reported. This systematic review and meta-analysis aims to synthesize the prevalence of prescription opioid misuse in the USA and examine the rate of transition to injection drug use and incident HCV in these new people who inject drugs (PWID).

**Methods/design:**

Eligible studies will include quantitative, empirical data including national survey data. Scientific databases will be searched using a comprehensive search strategy; proceedings of scientific conferences, reference lists, and personal communications will also be searched. Quality ratings will be assigned to each eligible report using the Newcastle-Ottawa Scale. Pooled estimates of incidence rates and measures of association will be calculated using random effects models. Heterogeneity will be assessed at each stage of data synthesis.

**Discussion:**

A unique typology of drug use is emerging which is characterized by antecedent prescription opioid misuse among PWID. As the epidemic of prescription opioid misuse matures, this will likely serve as a persistent source of new PWID. Persons who report a recent transition to drug injection are characterized by high rates of HCV seroincidence of 40 per 100 person years or higher. Given the potential for the persistence and escalation of the consequences of prescription opioid misuse in the USA, there is a critical need for synthesis of the current state of the epidemic in order to inform future public health interventions and policy.

**Systematic review registration:**

PROSPERO CRD42014008870.

## Background

The production, prescription, and consumption of prescription opioids—specifically pharmaceutical opioid analgesics designed and used for the treatment of severe or chronic non-cancer pain—have increased rapidly over the past decade [1-3]. Since 1999, the number of prescriptions for opioid analgesics rose by 350% in the USA [4]. The upsurge in the availability and use of prescription opioids has been linked to their widespread misuse [5]. High rates of misuse have been reported in parts of Europe, Australia, and North America; recent data shows that prevalence of prescription opioid misuse has also emerged, and is rising, in parts of Asia and Africa [6]. Although Americans constitute only 4.6% of the world's population, they consume 80% of the global opioid supply [7,8]. As of 2012, prescription opioids surpassed marijuana as the most misused substance in the USA, where prescription opioid misuse has recently been classified as an epidemic [9].

The rapid rise of prescription opioid misuse in the USA has been most dramatic among young people [10-12]. The incidence rate of prescription opioid misuse for 12–25-year olds was relatively stable and low from 1979 through the early 1990s; incidence began to rise in 1994 from 12–13 per 1,000 persons to 30–50 per 1,000 in 2001 among those 12–25-year olds [13,14]. In 2010, 11%, or 3.4 million, 18- to 25-year olds were reporting prescription opioid misuse [15-17]. A recent US survey of suburban adolescents and early adults showed that one-third reported misuse of prescription opioids by the age of 21 [16].

There is significant morbidity and mortality associated with the misuse of prescription opioids, including unintentional overdose. Overdose deaths due to prescription opioids quadrupled between 1999 and 2007 [18]. In 2008, overdose deaths due to misuse of prescription opioids ranged from 5.5–27 per 100,000 persons depending on geographic location [19]. Overdose due to prescription opioids now exceeds those due to heroin and cocaine combined [20]. Emergency departments have also seen a dramatic rise in visits related to prescription opioid use where visits have more than doubled to nearly half a million since 2004 [21,22]. Annual direct health care costs due to prescription opioid misuse have been estimated to be $55.7 billion [23].

A less well-documented phenomenon has been the pathway from prescription opioid misuse to injection of both synthetic opioids and heroin [13]. Both qualitative and survey data point to a trend of antecedent prescription opioid misuse among new people who inject drugs (PWID) [24-26]. As drug dependence and tolerance increase, many—perhaps 10%–20%—who are misusing these prescription drugs will escalate to injection [27,28]. In a study by Mars et al., young adult heroin users were more likely to have misused prescription opioids prior to transitioning to heroin injection at rates much higher than their older counterparts [13].

It is among these new PWID that clusters of hepatitis C (HCV) infection have been observed [3,29]. As the epidemic of misuse of prescription opioids matures, it will likely serve as a persistent source of new PWID, a group characterized by high rates of HCV seroconversion of 40 per 100 person years or higher [30,31]. Given the high and sustained availability of prescription opioids via illicit and prescription drug markets, and the risk of transition to drug injection among those using prescription opioids, outbreaks of acute HCV are likely to continue. The threat of emergent and persistent HCV infection among this growing risk population could cause an escalation in national prevalence and incidence. The purpose of this review is to synthesize the research reporting on the consequences of the recent upsurge in the misuse of prescription opioids in the USA.

## Methods/design

### Design and scope

This study will consist of a systematic review and meta-analysis of the prevalence of prescription opioid misuse, rates of transition from prescription opioid misuse to first-time injection drug use and the incidence of HCV among these new PWID. This review will characterize the prevalence of prescription opioid misuse in US settings from both non-probability and survey data and calculate pooled and subgroup-specific rates of transition to drug injection, and calculate pooled and subgroup-specific HCV incidence rates in those who initiate drug injection using random effects meta-regression models.

### Criteria for considering studies

#### Inclusion and exclusion criteria

Published and unpublished data reports, personal communications, dissertations, abstracts, conference presentations, and book chapters are eligible for inclusion in the study. Data reports will be included if they became available from 1 January 1990 through 30 June 2014. Studies will be included if they reported on prevalence or incidence of prescription opioid misuse or rates of transition from prescription opioid misuse to first-time injection drug use and HCV incidence among those recently transitioned. Data reports where recent initiates of drug injection are a subset, and not the main focus of study, will be eligible for inclusion in the review. Conference abstracts will be included if sufficient data were reported. Case reports or case series where the total number of cases was fewer than 10 will be excluded. Case-control, cohort, and cross-sectional study designs will be eligible for inclusion in the systematic review. Studies not eligible for inclusion include randomized controlled trials of interventions intended to alter the outcomes studied in this review.

#### Outcome measures

There are three main outcomes of this systematic review: prescription opioid misuse prevalence and incidence, rates of transition to drug injection among prescription opioid misusers, and HCV seroconversion among new PWID with antecedent prescription opioid misuse. Definitions of what constitutes a newly transitioned PWID used in the data reports will be recorded; higher quality ratings will be given to reports that confirm drug injection naivety among those defined as being new PWID and that provide a time frame for when initiation into injection drug use occurred.

Rates of acute HCV infection or seroconversion will be included. We define subjects with ‘HCV seroconversion’ or ‘acute HCV’ as those who have been screened and tested positive for HCV antibody (serology) or RNA within 12 months of previously testing negative. We adopted the European AIDS Treatment Network (European NEAT) Acute Hepatitis C Infection Consensus Panel criteria [32]:

*Preferred criteria* - seroconversion or positive HCV RNA and a documented negative HCV RNA or negative HCV antibody in the previous 12 months.

*Alternative criteria* - includes positive HCV RNA and an elevated ALT with or without other clinical signs of hepatitis.

Data reports using the preferred criteria will be given higher quality ratings than those that use the alternative criteria. An initial examination of the available literature suggested that there would be very few reports of HCV infection determined by laboratory testing. Thus, we will include studies that rely on self-reported HCV status but they will receive lower quality scores and be subject to greater scrutiny in analysis and interpretation.

#### Exposure measures

The primary exposure of interest to this systematic review is prescription opioid misuse. In response to the use of varied and idiosyncratic definitions and measurements of prescription opioid misuse in the literature, there has been a collective effort toward the adoption of a standardized operational definition of misuse. In 2006, the National Institute of Drug Abuse (NIDA) officially defined prescription opioid ‘abuse’ as follows: ‘[opioid abuse includes any] intentional use of opioids outside of a physician's prescription for a bona fide medical condition, excluding accidental misuse’ [1]. The systematic review will record the operational definitions of misuse used in the studies (e.g., any nonmedical use of a PO; any use of a PO; taking a PO for the way it makes one feel). We anticipate that the ‘dose’ of PO exposure in terms of frequency, recency, and amount will be related to the likelihood of transitioning to injection drug use; this will be examined in the analysis. For example, the rate of transition to injection would be compared between studies defining PO misuse as any nonmedical use in the participant's lifetime (‘low’ exposure dose) and studies that report daily PO misuse during a recent period (‘high’ dose).

#### Search strategy

A medical librarian was consulted regarding the search methods. Automated searches of published literature (title, abstract, and keywords) will be conducted using the following electronic databases: PubMed, OVID, EMBASE, Web of Knowledge, and PsycINFO. Search terms included those related to prescription opioid misuse, initiation of injection drug use, and HCV incidence among new PWID.

#### Screening and data collection

As an initial screening step, the title and abstract of data reports retrieved via the search will be read by the study's Principal Investigator (PI; HH) and the Project Director (PD; AEJ); abstracts with any mention of the main outcomes of interest will be considered for inclusion and the full text article will be retrieved. A pilot study will be carried out to test and refine procedures for screening and data abstraction. Discrepancies between the pilot screening results will be discussed and the protocol will be revised to clarify procedures. This process will be repeated until consensus is reached.

Abstracts and full-text articles obtained via the search strategy will be imported into Endnote X6 (Reuters) and duplicates will be deleted. Reasons for exclusion will be recorded. Relevant data will be abstracted onto a paper instrument adapted from those used in a series of prior systematic reviews led by the PI. Once this is complete, data will be entered into a Microsoft Access database. Data to be abstracted will include: citation information; study years and locations; study design; methods and sites used to recruit study participants; prevalence and incidence of prescription drug misuse, and factors associated with both measures; rates of transition to injection drug use and factors associated with transition; HCV seroincidence among new PWID reporting previous prescription opioid misuse; factors associated with HCV seroconversion; and other relevant demographic characteristics of the study sample.

### Quality assurance

The PD and a Master's-level trained epidemiologist, both with expertise in research methodology, HCV, drug use, and systematic reviews and meta-analytic methods, and a research assistant with expertise in research methods and training on HCV and drug use, will carry out coding; the PD will review all coding forms to ensure completeness and accuracy of coding. During weekly staff meetings, any inconsistencies will be discussed and resolved. A written study manual was developed to guide the process and to record special cases and their resolution.

### Study quality and critical appraisal

In order to assess the quality of data reports, this synthesis will employ a quality rating procedure based on the Newcastle-Ottawa Scale (NOS) which assigns quality ratings to studies in relation to threats to internal validity (selection bias, misclassification of exposure or outcome, and confounding due to non-comparability of the groups being compared) [33]. Some types of bias will be addressed through screening of reports for eligibility. Eligibility screening also will address potential misclassification of the outcome (e.g., acute or recent vs. chronic HCV infection). In addition to the NOS ratings, publication bias will be examined by comparing estimates between published and unpublished studies and by the use of funnel plots [34].

#### Selection bias

Selection bias has the potential to affect both case-control and cohort studies; the evaluation of selection bias in this synthesis will require the assessment of whether similar and adequate methods were employed to classify those who constituted cases and controls. In case-control studies, we will assess whether cases and controls arose from the same underlying population. Selection bias will be assessed in cohort studies in relation to whether the selection of the exposed cohort was related to the likelihood of any of the outcomes of interest (e.g., prescription opioid misuse).

#### Comparability

Quality assessment will also examine the comparability of cases to controls in case-control studies. In these studies, we will examine the use of matching or adjustment for confounding based on the differential distribution of factors among cases and controls in order to reduce biases. In cohort studies, we will assess whether the study adjusted for important differences across the exposed and unexposed cohorts. Adjusting for these differences is a critical factor in assessing the quality of the study's reporting of an association between assessing the exposure and the outcome.

#### Misclassification

Higher quality ratings will be given to data reports that provide an explicit definition of exposure and outcome. In case-control studies, classification of cases and controls with respect to exposure must be unbiased, and use of the same method of ascertaining exposure for cases and controls is preferred. In cohort studies, misclassification of outcome will be assessed in the quality ratings; for example, studies using the NEAT preferred definition of acute or recent HCV infection will be given higher scores.

### Data analysis

Aggregate (study-level) data will be used in this synthesis. Synthesis begins with the search for homogeneous subsets within sets of studies, followed by meta-analysis and calculation of summary estimates within the homogeneous subsets. Graphical and statistical analysis will be conducted using software designed specifically for meta-analysis. Variability in effects among the studies may reflect important differences, or confounding by other factors. Therefore, evidence of heterogeneity will be evaluated at each step in the analysis to distinguish between true variation of effects and heterogeneity due to other differences.

Data reports that present on HCV seroincidence among new PWID but did not inquire about previous experience injecting drugs (i.e., confirming that all new PWID were naïve to injection as a form of drug administration) will be analyzed separately. The reason for this is that we believe new PWID present unique risk factors for HCV acquisition [35-37].

Reports will also be analyzed by year of data collection and/or year of publication in order to examine whether there are changes in exposures or outcomes as the epidemic matures and as new policies are adopted.

Effect measures reported as hazard ratios, risk ratios, or relative risks will be transformed into odds ratios using standard methods. Meta-analysis and random effects meta-regression will be carried out. Meta-regression will be conducted to identify factors associated with variation in effect sizes (e.g., with higher versus lower effect sizes).

## Discussion

Pharmaceutical opioid misuse has been a long-standing public health problem in the USA [7,38,39]. However, overall misuse rates have historically been stable and relatively low-level in scope [13,38].

We anticipate that the majority of data reports retrieved will present prescription opioid misuse prevalence data from both non-probability samples (e.g., cohort studies) and household-based surveys (e.g., National Survey on Drug Use and Health). A casual examination of the literature also suggests that there are a substantial number of qualitative research studies on the topic that will not be eligible, but may provide important insights into the interpretation of the quantitative results.

Since the early 1990s, the annual number of prescriptions dispensed for opioid analgesics to treat non-cancer pain tripled reaching into the hundreds of millions and are now the most prescribed class of medications in the USA [40]. The average milligram prescribed per person rose from 74 to 369 between 1997 and 2007, an increase of over 400 percent [41]. Morbidity and mortality due to prescription opioid use in the form of accidental overdose and transition to riskier forms of drug use, rose dramatically in tandem. Given the potential for the persistence and escalation of the morbidity and mortality of prescription opioid misuse in the USA, there is a critically important role for a systematic review of this kind to inform future interventions and policy on this public health crisis.

## Competing interests

The authors declare that they have no competing interests.

## Authors’ contributions

AEJ contributed to the writing of the manuscript. AEJ, HH, and DDJ contributed to the conception and design and critical revision of the manuscript. All authors have read and approved the final version of the manuscript.
